# Profiling neuroinflammatory markers and response to nusinersen in paediatric spinal muscular atrophy

**DOI:** 10.1038/s41598-024-74338-z

**Published:** 2024-10-08

**Authors:** Qiang Zhang, Ying Hong, Chiara Brusa, Mariacristina Scoto, Nikki Cornell, Parth Patel, Giovanni Baranello, Francesco Muntoni, Haiyan Zhou

**Affiliations:** 1 Genetics and Genomic Medicine Research and Teaching Department, Great Ormond Street Institute of Child Health, University College London, London, UK; 2 School of Physical Education, Huangshan University, Huangshan, China; 3 Infection, Immunity and Inflammation Research and Teaching Department, Great Ormond Street Institute of Child Health, University College London, London, UK; 4 Developmental Neurosciences Research and Teaching Department, Great Ormond Street Institute of Child Health, The Dubowitz Neuromuscular Centre, University College London, London, UK; 5 NIHR Great Ormond Street Hospital Biomedical Research Centre, London, UK

**Keywords:** SMA, Neuroinflammation, Biomarker, CSF, Nusinersen, Biomarkers, Diseases, Medical research, Neurology

## Abstract

Neuroinflammation is an emerging clinical feature in spinal muscular atrophy (SMA). Characterizing neuroinflammatory cytokines in cerebrospinal fluid (CSF) in SMA and their response to nusinersen is important for identifying new biomarkers and understanding the pathophysiology of SMA. We measured twenty-seven neuroinflammatory markers in CSF from twenty SMA children at different time points, and correlated the findings with motor function improvement. At baseline, MCP-1, IL-7 and IL-8 were significantly increased in SMA1 patients compared to SMA2, and were significantly correlated with disease severity. After six months of nusinersen treatment, CSF levels of eotaxin and MIP-1β were markedly reduced, while IL-2, IL-4 and VEGF-A were increased. The decreases in eotaxin and MIP-1β were associated with changes in motor scores in SMA1. We also detected a transient increase in MCP-1, MDC, MIP-1α, IL-12/IL-23p40 and IL-8 after the first or second injection of nusinersen, followed by a steady return to baseline levels within six months. Our study provides a detailed profile of neuroinflammatory markers in SMA CSF. Our data confirms the potential of MCP-1, eotaxin and MIP-1β as new neuroinflammatory biomarkers in SMA1 and indicates the presence of a subtle inflammatory response to nusinersen during the early phase of treatment.

**Supplementary Information:**

The online version contains supplementary material available at 10.1038/s41598-024-74338-z.

## Introduction

Spinal muscular atrophy (SMA) is an autosomal recessive neurodegenerative disorder caused by mutations in the *survival motor neuron 1* (*SMN1*) gene^1^. This leads to a deficiency in SMN protein, resulting in motor neuron death, skeletal muscle atrophy and multiorgan abnormalities^2^. *SMN2* is a paralogous centromeric gene to *SMN1* that undergoes alternative splicing of exon 7 due to a cytosine to thymine variation, causing exon 7 skipping in 80–90% of *SMN2 *transcripts^3^. As a result of this splicing event, the *SMN2* gene cannot fully compensate for the loss of *SMN1* gene. However, the copy number of *SMN2 *gene correlates inversely with the clinical severity of the disease^4^. SMA patients are classified into four major subgroups based on the age of onset and clinical severity^5^. Type 1, 2 and 3 manifest during infancy and childhood, whereas type 4 has adult onset. Type 1 SMA (SMA1) is the most common and severe form of the disease in children.

Three SMN-enhancing therapies are currently available for SMA patients: the antisense drug nusinersen, AAV-mediated gene therapy onasemnogene abeparvovec, and the small molecule drug risdiplam^6–10^. These SMN-dependent treatments have provided unprecedented clinical improvements and have fundamentally altered the disease course^11^. Nusinersen was the first FDA-approved antisense oligonucleotide (ASO) for the treatment of SMA^12^. It is an 18-mer ASO with phosphorothioate 2’-O-methoxyethyl (MOE) chemistry, designed to target the intronic splicing silencer N1 (ISS-N1) in intron 7, thereby restoring proper pre-mRNA splicing of exon 7 in the endogenous *SMN2 *gene^13,14^. Nusinersen is administered intrathecally via lumbar puncture and is delivered at regular intervals. It has shown effectiveness in treating SMA patients and has generally been well-tolerated by the tens of thousands of patients who have received it in early clinical trials and real-world settings^6^. However, rare adverse events have been reported in the post-marketing phase, including communicating hydrocephalus^15^, aseptic meningitis and angioedema^16,17^.

Although SMA is primarily a lower motor neuron disease, it is widely recognized that SMN deficiency in multiple cell types and organs also contributes to its complex etiology. Immune dysregulation has been implicated in the pathogenesis of SMA, as demonstrated in transgenic mice and Drosophila model^18–22^. Even partial SMN deficiency in mild and intermediate Drosophila models can disrupt the innate immune system, resulting in hyperactivated neuroinflammation and subsequent neurodegeneration^22^. Recent studies have also shown evidence of peripheral immune system dysfunction and neuroinflammation in the central nervous system (CNS) of SMA patients, with improvements observed following nusinersen treatment^23–25^. Furthermore, increased activation of NK cells and CD8 + T cells within the CSF of SMA patients has been reported, suggesting potential immune cytotoxicity involvement in the CNS pathogenesis of SMA^26^. However, comprehensive data on the profile of neuroinflammatory markers in the CSF of SMA patients, both before and after nusinersen treatment, remain limited.

This study aims to investigate the neuroinflammatory marker profile in the CSF of SMA patients receiving repeated intrathecal nusinersen injections and to correlate these findings with clinical improvement in motor function. The study offers a comprehensive overview of baseline neuroinflammatory markers in the CSF of SMA1 and 2 patients, their response to nusinersen treatment, and identifies a subset of potential biomarkers associated with disease severity and therapeutic response. Our findings confirm the presence of baseline neuroinflammation in SMA patients, which can be transiently exacerbated during the loading phases of nusinersen by repeated administration, with improvements observed during the maintenance phase.

## Results

### Increased levels of neuroinflammatory markers in SMA1 patients compared to SMA2

**Fig. 1:**
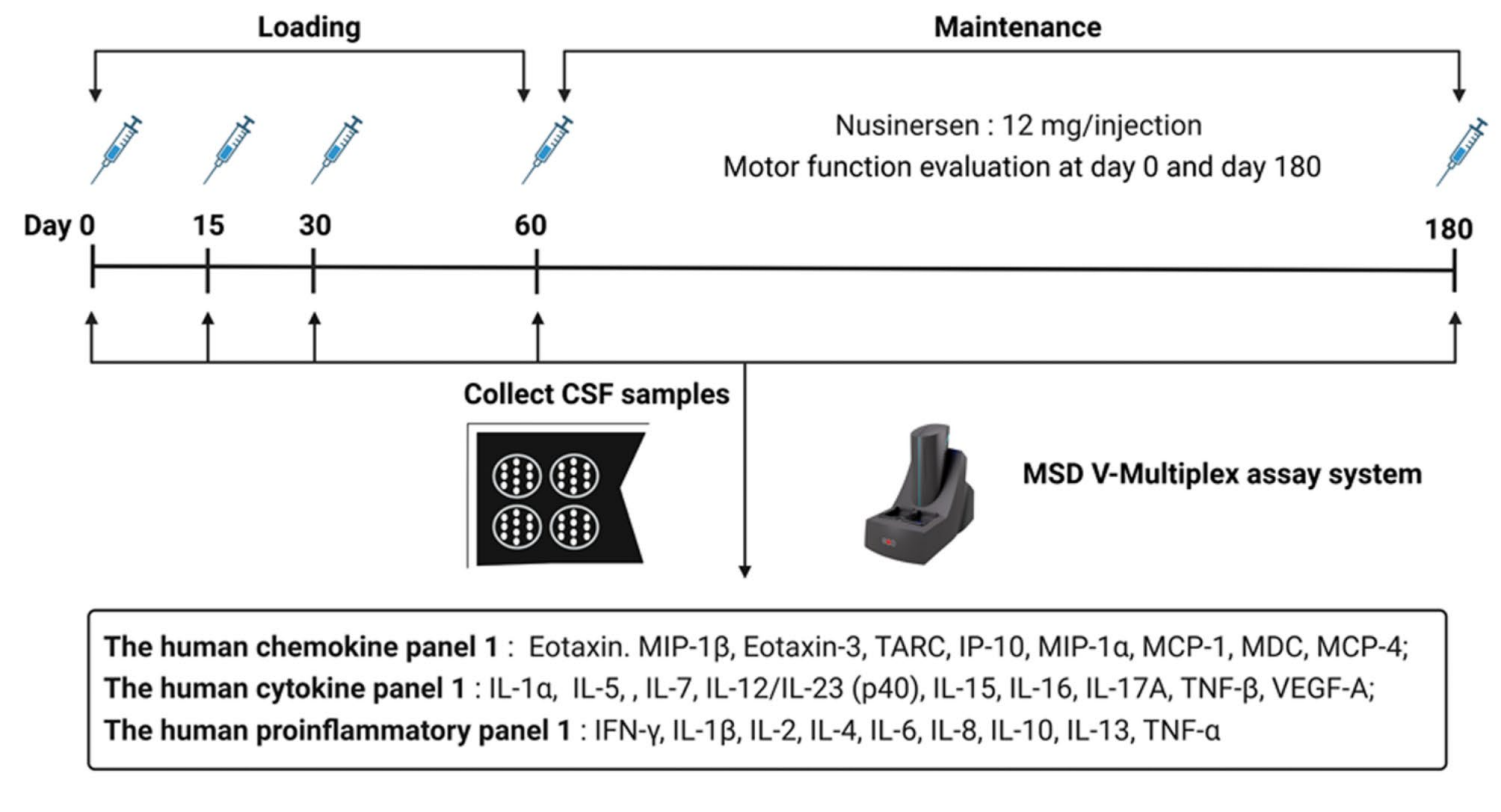
Illustration of experiment design. SMA patients received nusinersen at a dosage of 12 mg per dose, administered at day 0 (baseline), 15, 30, 60, and 180. CSF samples were collected at the same time. The levels of chemokines, cytokines and proinflammatory markers were measured using the appropriate panels from the Meso Scale Discovery (MSD) assay system.

We measured 27 neuroinflammatory markers in CSF samples from patients with SMA1 (*n* = 16; median age = 0.76 years, age range 0.18–1.67 years) and SMA2 (*n* = 4; median age = 1.79 years, age range 1.58–2.33 years) before and after the initiation of nusinersen treatment (Fig. 1; Table 1).

**Table 1:** Clinical data of the SMA patients involved in this study.

Patients ID	Gender	SMN2 copy number	SMA clinical type	Motor score at baseline	Motor score at 6 months after nusinersen treatment	Disease onset (year)	Age at treatment (year)
Patient-01	M	3	1c	CHOP-INTEND: 40/64	N/A	0.50	1.58
Patient-02	M	2	1b	CHOP-INTEND: 28/64	CHOP-INTEND: 39/64	0.12	0.33
Patient-03	F	2	1c	CHOP-INTEND: 29/64	CHOP-INTEND: 40/64	0.38	0.98
Patient-04	F	2	1b	N/A	CHOP-INTEND: 37/64	0.12	0.83
Patient-05	M	2	1b	N/A	CHOP-INTEND: 23/64	0.21	0.85
Patient-06	M	2	1b	N/A	N/A	0.13	0.89
Patient-07	F	2	1b	N/A	CHOP-INTEND: 43/64	0.10	0.24
Patient-08	M	2	1b	N/A	N/A	0.25	1.36
Patient-09	M	2	1b	CHOP-INTEND: 29/64	CHOP-INTEND: 44/64	0.24	0.35
Patient-10	F	2	1b	CHOP-INTEND: 23/64	CHOP-INTEND: 50/64	0.12	0.18
Patient-11	M	2	1b	N/A	CHOP-INTEND: 41/64	0.08	0.30
Patient-12	F	2	1b	CHOP-INTEND: 30/64	N/A	0.25	0.56
Patient-13	M	2	1b	CHOP-INTEND: 37/64	CHOP-INTEND: 46/64	0.08	1.67
Patient-14	F	2	1b	CHOP-INTEND: 22/64	CHOP-INTEND: 33/64	0.04	0.37
Patient-15	M	3	1b	CHOP-INTEND: 36/64	CHOP-INTEND: 43/64	0.08	1.02
Patient-16	F	3	2	RHS: 9/69	RHS: 15/69	0.58	1.58
Patient-17	M	3	2	RHS: 19/69	RHS: 17/69	1.54	2.33
Patient-18	F	2	1b	N/A	N/A	0.20	0.70
Patient-19	M	3	2	RHS: 2/69	RHS: 8/69	0.83	1.79
Patient-20	F	3	2	RHS: 11/69	RHS: 19/69	0.83	1.80

Presented are clinical data including gender, *SMN2* copy numbers, clinical subtypes, age at disease onset, age at first nusinersen dose, and motor scores assessed by CHOP-INTEND or RHS at baseline, and after 2 or 6 months of treatment. CHOP-INTEND, Children’s Hospital of Philadelphia Infant Test of Neuromuscular Disorders; RHS, revised Hammersmith score. N/A, not available.

**Fig. 2:**
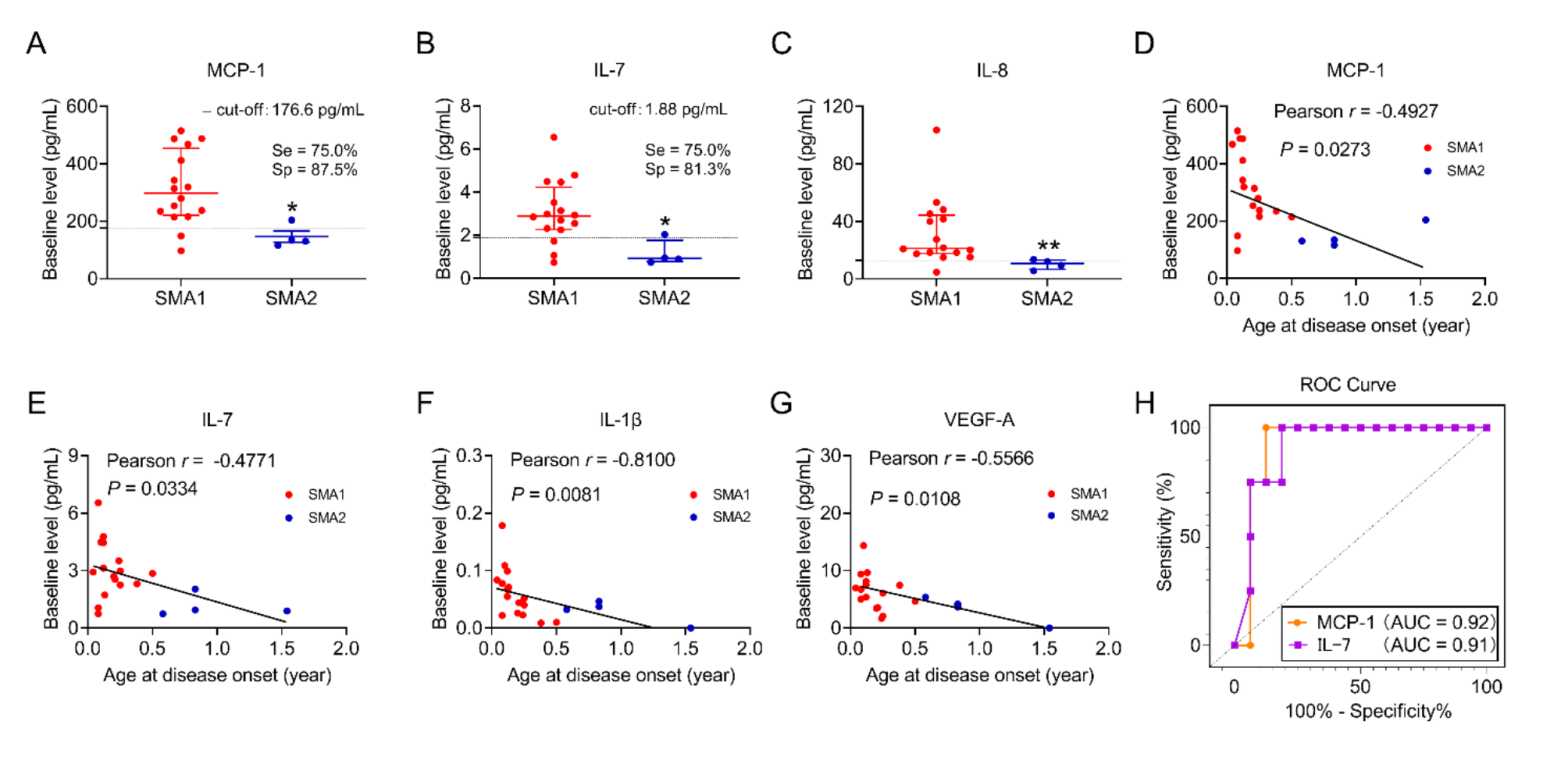
The CSF levels of MCP-1, IL-7 and IL-8 in SMA patients. Significantly increased levels of MCP-1 (A), IL-7 (B) and IL-8 (C) were observed in the CSF of SMA1 patients compared to SMA2 patients. Cut-off levels, along with the sensitivity (Se) and specificity (Sp) rates, are indicated. Pearson correlation analysis was performed to assess the correlation of the baseline levels of MCP-1 (D), IL-7 (E), IL-1β (F), and VEGF-A (G) with the age of onset. (H) ROC curve analysis for MCP-1 and IL-7 was conducted between SMA1 and SMA2 patients, and the area under the ROC curve (AUC) for both MCP-1 and IL-7 is indicated. *P < 0.05, **P < 0.01.

We compared the baseline levels of neuroinflammatory markers between SMA1 and SMA2 patients. Of the 27 markers analyzed, monocyte chemoattractant protein 1 (MCP-1), interleukin (IL)-7, and IL-8 were significantly elevated in SMA1 patients compared to those with SMA2 (MCP-1: SMA1 297.1 picograms per milliliter (pg/mL), SMA2 132.9 pg/mL, *P* = 0.020; IL-7: SMA1 2.9 pg/mL, SMA2 0.9 pg/mL, *P* = 0.021; IL-8: SMA1 21.4 pg/mL, SMA2 10.8 pg/mL, *P* = 0.005) (Fig. 2A, B, & C). No significant differences were detected in the remaining 24 markers (Supplementary Fig. 1 and supplementary Table 1).

We then examined the correlation between the baseline levels of these markers and the clinical severity of the patients. A Pearson’s correlation analysis was performed between the baseline levels of all 27 markers and the patients’ age at disease onset. The results indicated that the CSF levels of MCP-1, IL-7, IL-1β, and vascular endothelial growth factor (VEGF)-A were significantly and inversely correlated with the age at symptom onset (Fig. 2D, E, F & G). However, no significant correlation was found for the other 23 markers (Supplementary Fig. 2).

Given the significant differences in MCP-1 and IL-7 levels observed, we hypothesized that the CSF levels of MCP-1 or IL-7 may serve as potential biomarkers for disease severity. To test this, we conducted receiver operating characteristic (ROC) curve analysis to determine the optimal cut-off values for MCP-1 and IL-7 that could effectively differentiate between SMA1 and SMA2 patients^27^. The inflection point of the ROC curve, representing the optimal balance between sensitivity and specificity, indicated that a CSF level of MCP-1 below 176.6 pg/mL or IL-7 below 1.88 pg/mL could distinguish SMA1 from SMA2 patients with 75.0% sensitivity and 87.5% specificity for MCP-1, and 81.3% sensitivity for IL-7 (Fig. 2A, B & H). These findings suggest that MCP-1 and IL-7 levels in the CSF could serve as potential biomarkers associated with disease severity.

### The correlation between the changes in the levels of neuroinflammatory markers and the response of motor function after 6 months treatment of nusinersen

**Fig. 3:**
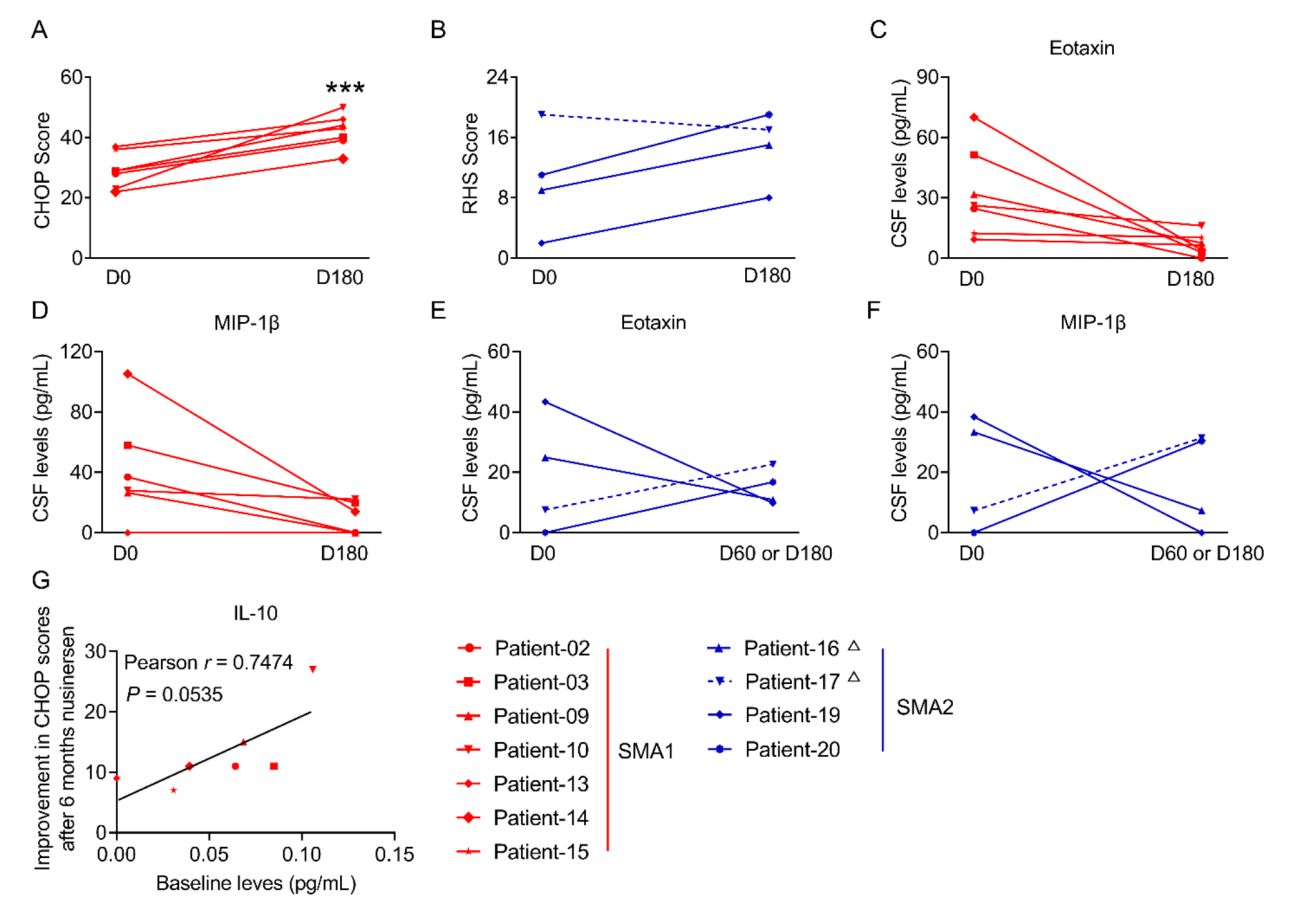
The changes in CSF levels of neuroinflammatory markers and in clinical motor scores in response to nusinersen treatment. The changes in CHOP motor scores in SMA1 patients (A) and RHS motor scores in SMA2 patients (B) were analyzed. The CSF levels of Eotaxin and MIP-1β were compared between baseline levels (D0) and after 6 months treatment of nusinersen (D180) in SMA1 patients (C&D) and SMA2 patients (E&F). Patients who showed motor function improvement are indicated with solid lines, while one SMA2 patient (Patient 17) with a decline in motor function is indicated with a dashed line. For patients No. 16 and 17, clinical motor scores were collected at 2 months of nusinersen treatment, as data at 6 months were unavailable. (G) A Pearson’s correlation study revealed that baseline levels of IL-10 were associated with the improvements in CHOP motor scores after 6 months of nusinersen treatment.

We evaluated the Children’s Hospital of Philadelphia Infant Test of Neuromuscular Disorders (CHOP-INTEND) or Revised Hammersmith Scale (RHS) scores (depending on age and type of SMA) before and after nusinersen treatment. Eleven patients had recorded motor scores at both baseline and 2 months (*n* = 2) or 6 months (*n* = 9) following treatment. Nusinersen treatment resulted in notable improvement in motor function for 10 SMA patients, classified as clinical responders (7 SMA1 and 3 SMA2), while one SMA2 patient was identified as a clinical non-responder, with an RHS score decreasing from 19 to 17 (patient No. 17 in Table 1) (Fig. 3A, B).

To assess whether the observed cytokine variations are associated with clinical response to therapeutic intervention, we analyzed the levels of 27 markers both at baseline and after nusinersen treatment. Among the markers examined, eotaxin and macrophage inflammatory protein (MIP)-1β consistently decreased in all 7 SMA1 patients who responded positively to nusinersen (Fig. 3C, D). However, this trend was not consistent among the four SMA2 patients. In particular, one SMA2 responder (patient No.20) showed increased levels of eotaxin and MIP-1β, a pattern similar to the non-responder (patient No.17) but divergent from the trend seen in SMA1 patients (Fig. 3E, F). No consistent trends were observed for the remaining 25 markers in either SMA1 or SMA2 patients (Supplementary Fig. 3). Additionally, no correlation was found between the baseline levels of eotaxin or MIP-1β and patient age (Supplementary Fig. 4A, B), ruling out the possibility that the reduction in SMA1 patients post-nusinersen was influenced by age.

Interestingly, a marginally significant correlation (*P* = 0.0535) was observed in SMA1 patients between the baseline IL-10 levels and changes in motor scores after 6 months of nusinersen treatment (Fig. 3G). This suggests that IL-10 may serve as a potential biomarker for predicting clinical improvement in motor function in response to nusinersen. No significant correlations were detected between other markers and motor function performance (Supplementary Fig. 5).

## The dynamic changes of neuroinflammatory markers in CSF in the first 6-month treatment of nusinersen

**Fig. 4:**
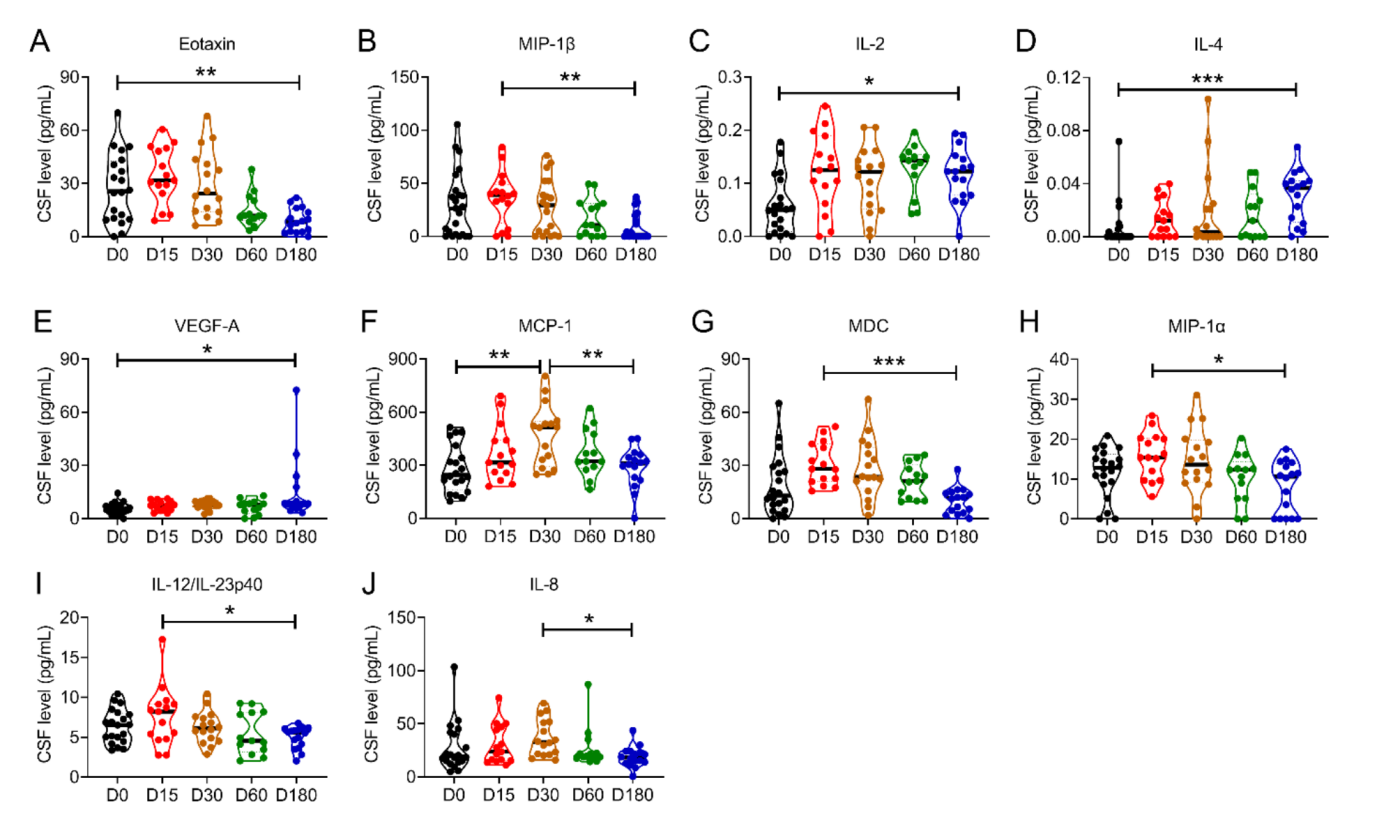
The CSF levels of neuroinflammatory markers in the first 6-month treatment of nusinersen. The CSF levels of Eotaxin (A), IL-2 (B), IL-4 (C), VEGF-A (D), MCP-1 (E), MDC (F), MIP-1α (G), MIP-1β (H), IL-12/IL-23p40 (I), and IL-8 (J) showed statistically significant dynamic changes between baseline (D0) and different time points (day 15, 30, 60 and 180) during the first 6 months of nusinersen treatment. Data were analyzed using One-way ANOVA followed by Turkey’s post-hoc or the Kruskal-Wallis’s test when the normal distribution assumption were met. In cases where normal distribution was not met, the Brown-Forsythe and Welch ANOVA were performed for multiple group comparisons. *P < 0.05, **P < 0.01, ***P < 0.001.

To assess the longitudinal impact of nusinersen on neuroinflammation in CSF, we measured all the 27 makers in the CSF samples collected from SMA patients at baseline and at 15, 30, 60 and 180 days after treatment (Supplementary Table 1). Bonferroni multiple comparisons analysis revealed a significant reduction in eotaxin (D0: 25.6 pg/mL, D180: 8.2 pg/mL, *P =* 0.0047; Fig. 4A) and MIP-1β (D0: 30.6 pg/mL, D15: 40.0 pg/mL, D180: 0 pg/mL, *P =* 0.007; Fig. 4B) after 180 days of nusinersen treatment. Conversely, significant increases were detected in IL-2 (D0: 0.05 pg/mL, D180: 0.12 pg/mL, *P =* 0.0185; Fig. 4C), IL-4 (D0: 0.00 pg/mL, D180: 0.04 pg/mL, *P =* 0.0007; Fig. 4D) and VEGF-A (D0: 5.4 pg/mL, D180: 8.3 pg/mL, *P =* 0.0361; Fig. 4E).

In addition, we observed a significant increase in MCP-1 levels at D30 during the loading phase of nusinersen, which then returned to baseline by D180 (D0: 246.0 pg/mL, D30: 514.4 pg/mL, *P =* 0.0017; D180: 310.1 pg/mL, *P =* 0.0052; Fig. 4H). Similar trends were detected in macrophage-derived chemokine (MDC) (D15: 28.0 pg/mL, D180: 11.6 pg/mL, *P =* 0.0003; Fig. 4G), MIP-1α (D15: 15.4 pg/mL, D180: 10.6 pg/mL, *P =* 0.0232; Fig. 4H), IL-12/IL-23p40 (D15: 8.2 pg/mL, D180: 5.6 pg/mL, *P =* 0.028; Fig. 4I), and IL-8 (D30: 32.8 pg/mL, D180: 18.1 pg/mL, *P =* 0.024; Fig. 4J), which were transiently increased between Days 15 and 30 after nusinersen treatment and subsequently declined (Supplementary Table 1). No significant changes were observed in IL-1α, IL-1β, IL-5, IL-6, IL-7, IL-10, IL-13, IL-15, IL-16, IL-17 A, Eotaxin-3, tumor necrosis factor beta (TNF)-α, TNF-beta, thymus and activation-regulated chemokine (TARC), MCP-4, IP-10 and interferon (INF)-γ in CSF at any time points when compared to baseline (Supplementary Fig. 6 and Supplementary Table 1).

## Discussion

Neuroinflammation is an emerging clinical feature reported in SMA patients^28,29^. Elevated neuroinflammatory cytokines in the CSF of untreated SMA patients and their responses to nusinersen treatment have been sporadically documented^24,25^. However, further validation in larger patient cohorts using different analytical platforms is needed to confirm these findings.

In this study, we conducted a comprehensive profiling of 27 inflammatory markers in CSF samples collected from 20 pediatric SMA patients during the early course of nusinersen treatment. By analyzing the baseline levels of these inflammatory markers, we identified a significant difference in MCP-1 levels between SMA1 (297.1 pg/mL) and SMA2 (132.9 pg/mL) patients (Fig. 2A). Furthermore, a reverse correlation was observed between MCP-1 baseline levels and the age of disease onset (Fig. 2D). The potential of MCP-1 as a neuroinflammatory biomarker of SMA clinical severity is further supported by ROC curves analysis, where a CSF level of 176.6 pg/mL for MCP-1 can distinguish over 87% of SMA1 patients from SMA2 (Fig. 2H). Similar findings have been reported in two recent studies on SMA patients^24,25^. In Scheijmans’ study of 38 children with SMA, MCP-1 baseline levels were highest in SMA1 (1123.08 pg/mL), followed by SMA2 (737.93 pg/mL) and SMA3 (699.33 pg/mL). Similarly, Nuzzo et al. reported the highest MCP-1 levels in SMA1 (259.27 pg/mL), followed by SMA2 (199.42 pg/mL) and SMA 3 (163.54 pg/mL) in their cohort of 48 paediatric SMA patients^24^. While the absolute MCP levels varied across studies, likely due to differences in quantification methods, the consistent trends suggest MCP-1 as a promising neuroinflammatory biomarker for disease severity in SMA.

Interestingly, both Scheijmans and Nuzzo’s studies reported an increase in MCP-1 levels over time in nusinersen-treated SMA2 and/or SMA3 patients, but not in SMA1 patients. It is important to note that MCP-1 expression is also influenced by age^30,31^, and thus age should be considered when interpreting the reported increase in SMA2 and SMA3 over a 10-month period (from baseline to the 5th injection)^25^. In our study, which focused on the first 6 months of nusinersen treatment, we observed a significant increase in MCP-1 levels after 1 month of treatment. However, these levels gradually decreased and returned to baseline levels after 6 months. We hypothesize that this transient increase in MCP-1 is probably due to an acute innate immune response in the CNS to the repeated intrathecal injections of nusinersen during the loading phase, rather than being related to the therapeutic effect of the drug. Indeed, neither our study nor the previous studies demonstrated a correlation between MCP-1 levels and motor score outcomes, suggesting that MCP-1 may not serve as a reliable biomarker of therapeutic response to nusinersen.

IL-7 is another interesting neuroinflammatory marker identified in our study. Similar to MCP-1, IL-7 also demonstrated an inverse correlation with SMA severity. In Nuzzo’s study, IL-7 levels decreased after 10 months of nusinersen treatment^24^. However, in our study, no significant changes in IL-7 were observed after 6 months of treatment (Supplementary Fig. 6K). This difference is probably attributable to the shorter duration of treatment in our study, suggesting the need for further longitudinal studies to better understand the potential role of IL-7 in SMA and its response to nusinersen.

In the seven SMA1 patients with motor function scores available at both baseline and six months post-treatment, changes in eotaxin and MIP-1β levels may serve as biomarkers for motor function improvement following nusinersen treatment (Fig. 3). We excluded age as a confounding factor in this study (Supplementary Fig. 4). The reduction in eotaxin levels in response to nusinersen has also been observed in Nuzzo’s study, where a significant decrease in eotaxin in the CSF was detected after 10 months of treatment^24^. This finding aligns with our results, which show a reduction in eotaxin after 6 months of treatment (Fig. 4A). The consistent findings from both independent studies suggest that eotaxin may be a promising biomarker for the clinical response of SMA1 patients to nusinersen.

In our study we found that MIP-1β levels were reduced after 6 months of nusinersen treatment in all SMA1 patients (Fig. 3D) but not in SMA2 patients (Fig. 3F). In contrast, Nuzzo’s study reported divergent findings, where MIP-1β levels in the CSF did not change in SMA1 patients but increased in SMA2 patients after 302 days of nusinersen treatment^24^. The variance in MIP-1β data between these two studies indicates that further investigation into this cytokine’s role in SMA patients is necessary.

IL-10 was previously reported in Scheijmans study, where it was suggested that its elevated levels may be associated with increased leukocyte counts^25^. Additionally, Bonanno et al. demonstrated that SMA patients with higher serum IL-10 levels after six months of nusinersen treatment exhibited improved motor function score^23^. Consistent with Bonanno’s findings, our study also suggests that baseline IL-10 levels in the CSF may serve as a predictor of motor function improvement following nusinersen treatment. Specifically, a higher baseline level of IL-10 in CSF correlated with greater motor function improvement after six months of treatment (Supplementary Fig. 5). Indeed, as an anti-inflammatory cytokine, IL-10 plays a crucial role in modulating immune response by suppressing the activity of T cells, NK cells, and macrophages, thus regulating tissue inflammation^32,33^. Its neuroprotective role in motor neuron diseases has been supported by recent studies, including one in a mouse model of amyotrophic lateral sclerosis (ALS). In that study, intramuscular administration of IL-10 improved motor performance in ALS mice by enhancing satellite cells and the pro-regenerative activity of macrophages, leading to delayed muscle atrophy and motor neuron loss^34^. Given these findings, further investigation into the protective role of IL-10 in SMA, as well as its potential as a predictive biomarker of response to nusinersen, is worthy to explore.

In this study, we observed a transient neuroinflammatory response in the CSF of SMA patients following intrathecal nusinersen administrations during the loading phase. Fluctuations in several markers were detected at various time points, including transient increases in MCP-1, MDC, MIP-1α, IL-12/IL-23p40 and IL-8, which then returned to baseline levels (Fig. 4). However, the increases in IL-2, IL-4 and VEGF-A at the six-month mark in our study contrast with Nuzzo’s findings, where IL-2, IL-4 and VEGF were decreased in SMA1 patients after 10 months of treatment^24^. It is possible that the increases in these markers at six months were transient and may reduce over the longer term of treatment. Indeed, Nuzzo’s study also reported the transient and modest induction of pro-inflammatory cytokines (IL-8, G-CSF, MCP-1, MIP-1 α and MIP-1β) in the CSF of SMA2 and SMA3 patients, which were not linked to any clinical findings. However, longitudinal studies in patients undergoing long-term nusinersen treatment remains necessary for further investigation. Additionally, when compared our findings with a recent transcriptomic study investigating off-target effects of ISS-N1-targeting ASO in fibroblasts from SMA patients^35^, no overlapping genes were identified between the two studies. This suggests that the neuroinflammatory markers observed in our study are unlikely to be a result of any potential off-target effects of the ASO drug.

While our study provides important insights, it has some limitations. One limitation is the relatively small number of SMA patients, particularly in the group with milder SMA. This may partly explain the discrepancies observed between our findings and those from other studies. There was also a lack of CSF cytokine data from age-matched healthy children, which would have enabled direct comparisons with SMA patients.

In summary, our study presents a comprehensive profile of neuroinflammatory markers in the CSF of pediatric SMA patients treated with nusinersen. We identified MCP-1 as a potential marker of disease severity, and eotaxin and MIP-1β as markers of response to nusinersen, aligning with findings from previous studies. Our results also suggest a transient pro-inflammatory response to nusinersen in the CSF during the initial treatment period, which subsides as treatment progresses. The confirmation of neuroinflammatory cytokines in SMA, along with their response to nusinersen, not only highlights new biomarkers but also suggesting neuroinflammation as a potential contributor to SMA. These new findings may lead to further investigations in therapeutic intervention, improving our grasp of the complex pathophysiology of SMA.

## Methods

### Patient cohort

Twenty patients, comprising sixteen with SMA1 and four with SMA2, who received intrathecal administration of nusinersen, were included in this study^7^. CSF samples were collected at baseline and at various time points, following the standard clinical protocol. All samples were stored at the MRC Centre for Neuromuscular Diseases Biobank in London (REC reference number 06/Q0406/33, http://www.cnmd.ac.uk). The study received ethical approval from the Berkshire Research Ethics Committee (REC reference 05/MRE12/32), and informed consent was obtained from all patients or their legal guardians. All experiments were conducted in accordance with relevant guidelines and regulations.

All patients had a confirmed genetic diagnosis of SMA, with a homozygous genomic deletion in *SMN1* gene and two or three copies of *SMN2* gene. The patients’ ages ranged from 0.18 to 2.33 years. A summary of the patients’ clinical information is provided in Table 1.

### Motor function evaluation

Clinical assessments of patients were conducted by expert physiotherapists at both baseline and 180 days after the initiation of nusinersen treatment. Patients with SMA1 were assessed using the CHOP-INTEND^36^, consisting of 16 items designed to evaluate motor function in infants with muscle weakness. Each item is scored on a scale from 0 (no response) to 4 (complete response), resulting in a total score that ranges from 0 to 64. Patients with SMA type 2 were assessed using the RHS^37^, consisting of 36 items designed to capture motor function from very weak SMA2 patients to stronger SMA3 patients. The maximum score on the RHS is 69.

### CSF sample collection

CSF samples were collected at baseline (day 0, D0), day 15 (D15, ~ 2 weeks), day 30 (D30, ~ 1 month), day 60 (D60, ~ 2 months), and day 180 (D180, ~ 6 months) following the start of nusinersen treatment (Fig. 1). During each injection, approximate 3 mL of CSF was drawn before the administration of 5 mL of nusinersen. The collected samples were then aliquoted and stored at -80 °C for subsequent analysis.

### Quantification of neuroinflammatory markers by MSD V-Plex assay

The levels of cytokines, chemokines, and proinflammatory markers in CSF samples were measured using the V-PLEX neuroinflammation panel kits by Meso Scale Discovery (MSD) (Fig. 1). The human cytokine panel 1 included IL-1α, IL-5, IL-7, IL-12/IL-23p40, IL-15, IL-16, IL-17 A, TNF-β, VEGF-A; The human chemokine panel 1 included Eotaxin, MIP-1b, Eotaxin-3, TARC, IP-10, MIP-1α, MCP-1, MDC, MCP-4; The human proinflammatory panel 1 included IFN-γ, IL-1β, IL-2, IL-4, IL-6, IL-8, IL-10, IL-13, TNF-α.

All assays were performed in accordance with the manufacturer’s instructions (MSD K15210D-1). In brief, diluted CSF samples and reference standards were incubated with anti-human IgG antibodies, and plates were read using the MESO^®^ QuickPlex SQ 120 reader. Data were analyzed using the Discovery Workbench version 4.0 software, and concentrations of the parameters was presented in pg/mL.

### Statistical analysis

Data on CSF levels of neuroinflammatory biomarkers were analyzed using the Shapiro-Wilk test to assess normal distribution. For significance comparisons between two groups, we employed either an unpaired t-test or the Mann-Whitney test. When comparing three or more groups, we utilized One-way ANOVA followed by Tukey’s post-hoc test or Kruskal-Wallis test, depending on whether the normal distribution assumption was met. In cases where normal distribution assumption was not satisfied, the Brown-Forsythe and Welch ANOVA tests were applied for multiple-group comparisons. Continuous variables were represented as medians with interquartile ranges (IQR). Pearson’s correlation analysis was used to examine correlations between variables. To evaluate MCP 1 and IL-7 as potential markers distinguishing SMA1 from SMA2, we conducted ROC curve analysis. Statistical significance was set at *P < 0.05, ** *P* < 0.01, ***P < 0.001. All statistical analyses were performed using GraphPad Prism 9.0 software.

Presented are clinical data including gender, SMN2 copy numbers, clinical subtypes, age at disease onset, age at first nusinersen dose, and motor scores assessed by CHOP-INTEND or RHS at baseline, and after 2 or 6 months of treatment. CHOP-INTEND, Children’s Hospital of Philadelphia Infant Test of Neuromuscular Disorders; RHS, revised Hammersmith score. N/A, not available.

## Electronic supplementary material

Below is the link to the electronic supplementary material.

Supplementary Material 1

## Data Availability

All data generated or analysed during this study are included in this published article and its supplementary information files.
