# Supplementary material for: Profiling neuroinflammatory markers and response to nusinersen in paediatric spinal muscular atrophy
Source: Sci Rep. 2024 Oct 8;14:23491. doi: 10.1038/s41598-024-74338-z (PMC11461652; doi:10.1038/s41598-024-74338-z)
Supplement: Supplementary file 1 — Supplementary Material 1 [file 41598_2024_74338_MOESM1_ESM.docx]

**Supplementary Figures, legends and Table**


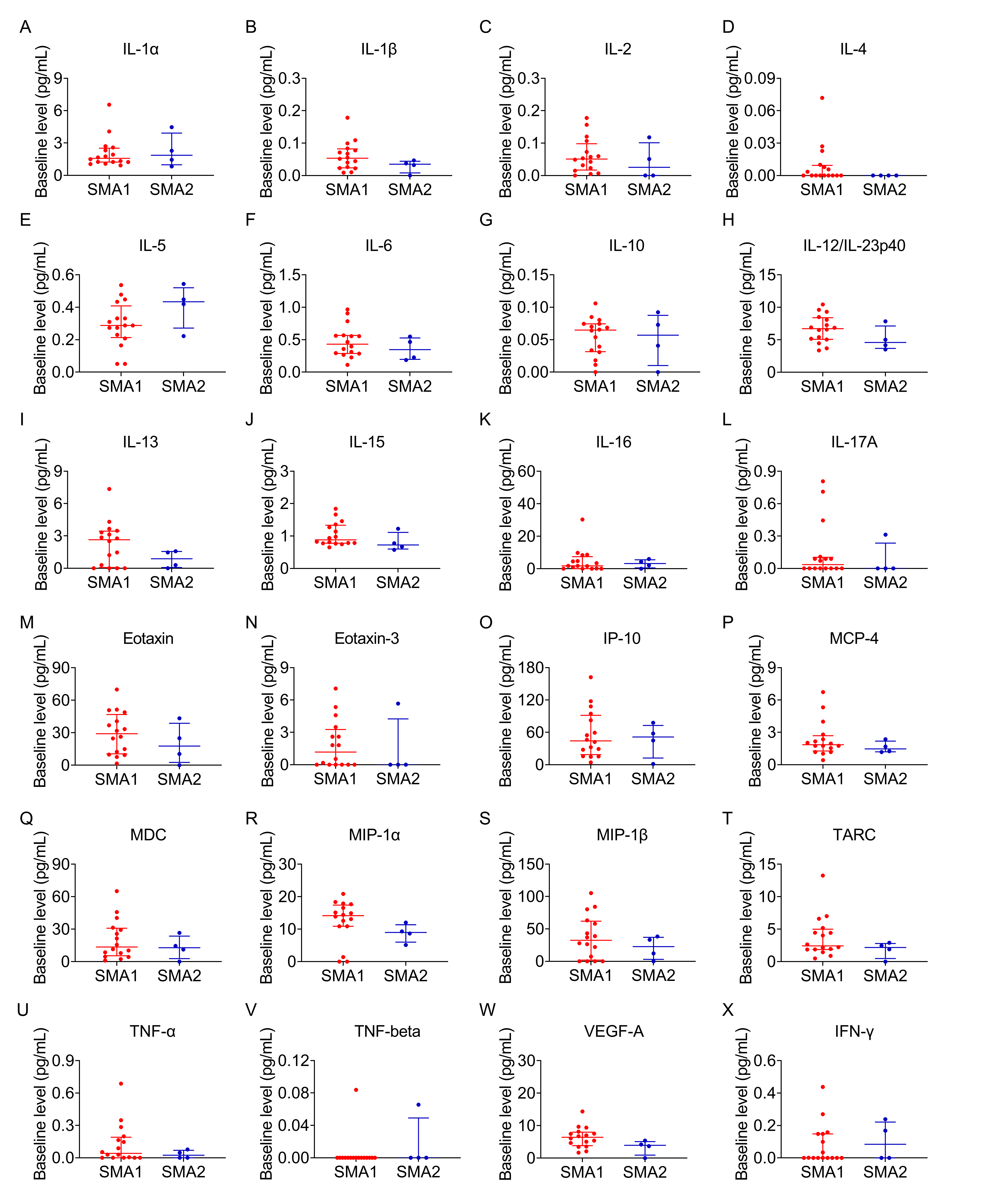


**Supplementary Figure 1.** Baseline CSF levels of neuroinflammatory markers were measured using MSD assays, including IL-1α (A), IL-1β (B), IL-2 (C), IL-4 (D), IL-5 (E), IL-6 (F), IL-10 (G), IL-12/IL-23p40 (H), IL-13 (I), IL-15 (J), IL-16 (K), IL-17A (L), Eotaxin (M), Eotaxin-3 (N), IP-10 (O), MCP-4 (P), MDC (Q), MIP-1α (R), MIP-1β (S), TARC (T), TNF-α (U), TNF-beta (V), VEGF-A (W), and INF-γ (X). No statistically significant differences were observed between SMA1 and SMA2 patients for these markers.


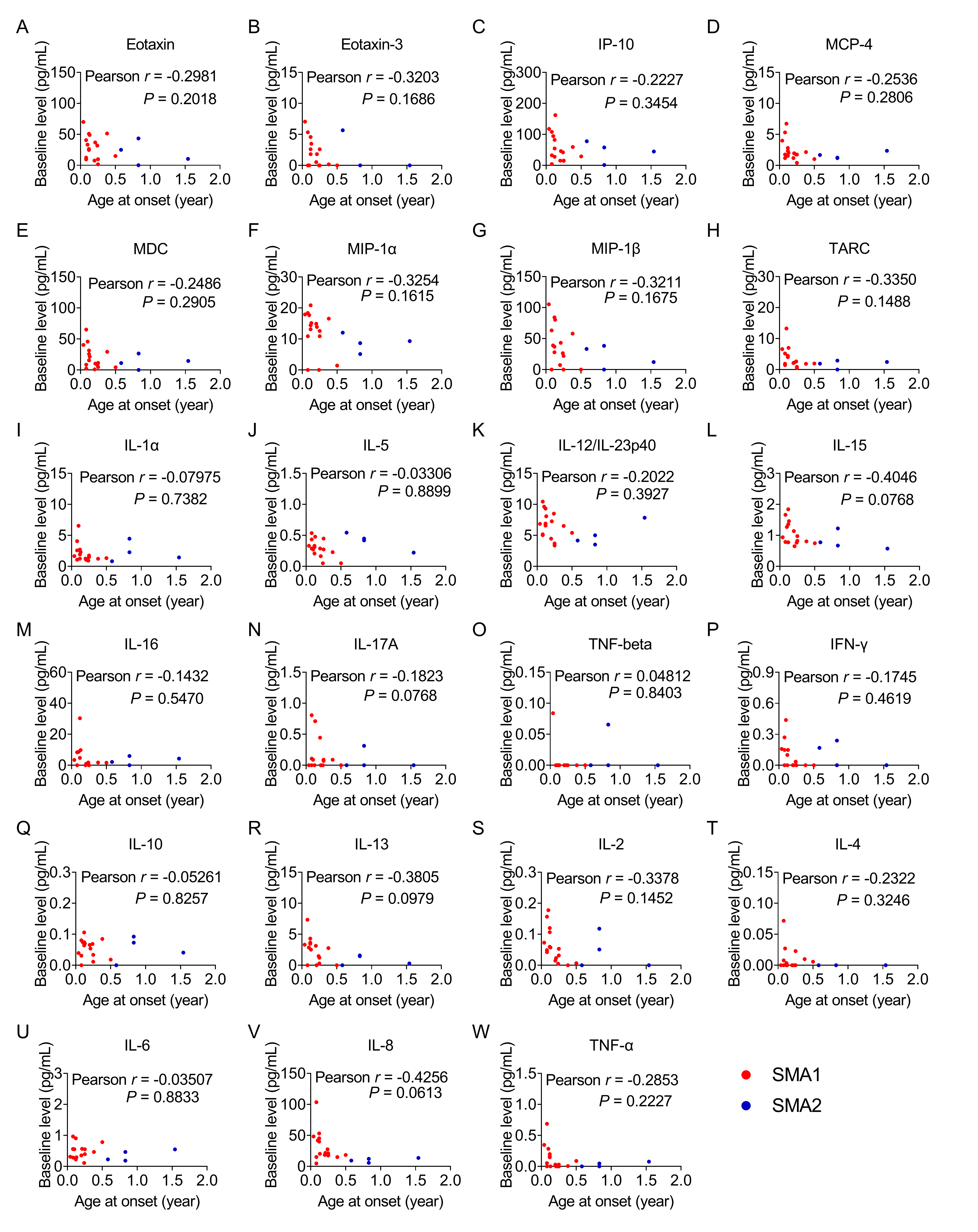


**Supplementary Figure 2.** Pearson correlation analysis was conducted to assess the relationship between the baseline levels of various neuroinflammatory markers and age at onset, including Eotaxin (A), Eotaxin-3 (B), IP-10 (C), MCP-4 (D), MDC (E), MIP-1α (F), MIP-1β (G), TARC (H), IL-1α (I), IL-5 (J), IL-12/IL-23p40 (K), IL-15 (L), IL-16 (M), IL-17A (N), TNF-beta (O), IFN-γ (P), IL-10 (Q), IL-13 (R), IL-2 (S), IL-4 (T), IL-6 (U), IL-8 (V), and TNFα (W). No statistically significant correlations were identified for any of these markers.

**Supplementary Figure 3.** Changes in the CSF levels of neuroinflammatory biomarkers before and after six months of nusinersen treatment are presented for Eotaxin-3 (A), IP-10 (B), MCP-1 C(), MCP-4 (D), MDC (E), MIP-1α (F), TARC (G), IL-1α (H), IL-5 (I), IL-7 (J), IL-12/IL-23p40 (K), IL-15 (L), IL-16 (M), IL-17A (N), TNF-beta (O), VEGE-A (P), IFN-γ (Q), IL-10 (R), IL-13 (S), IL-1β (T), IL-2 (U), IL-4 (V), IL-6 (W), IL-8 (X) and TNFα (Y). The clinical non-responder, patient No.17, is highlighted with a dashed line.


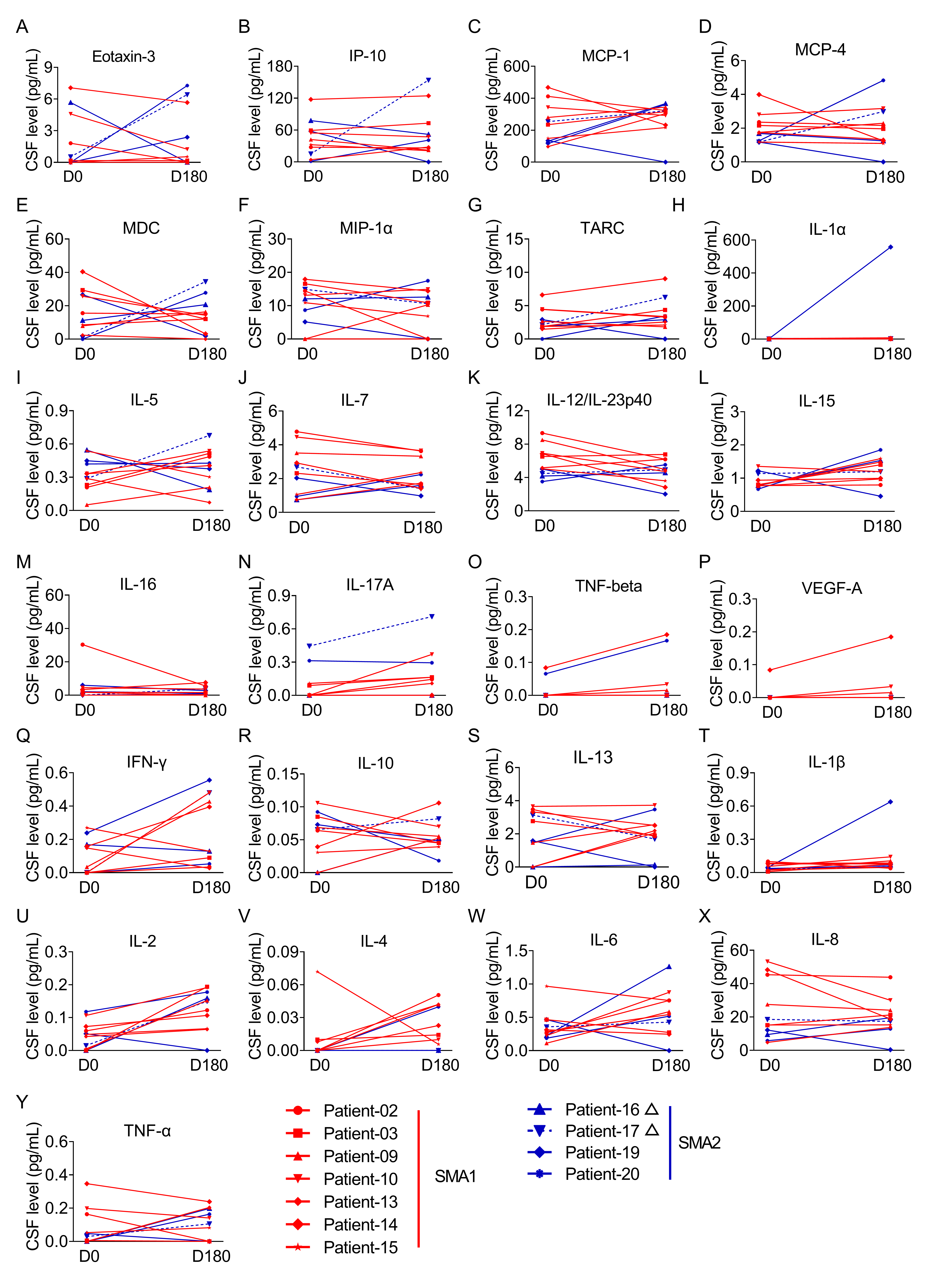

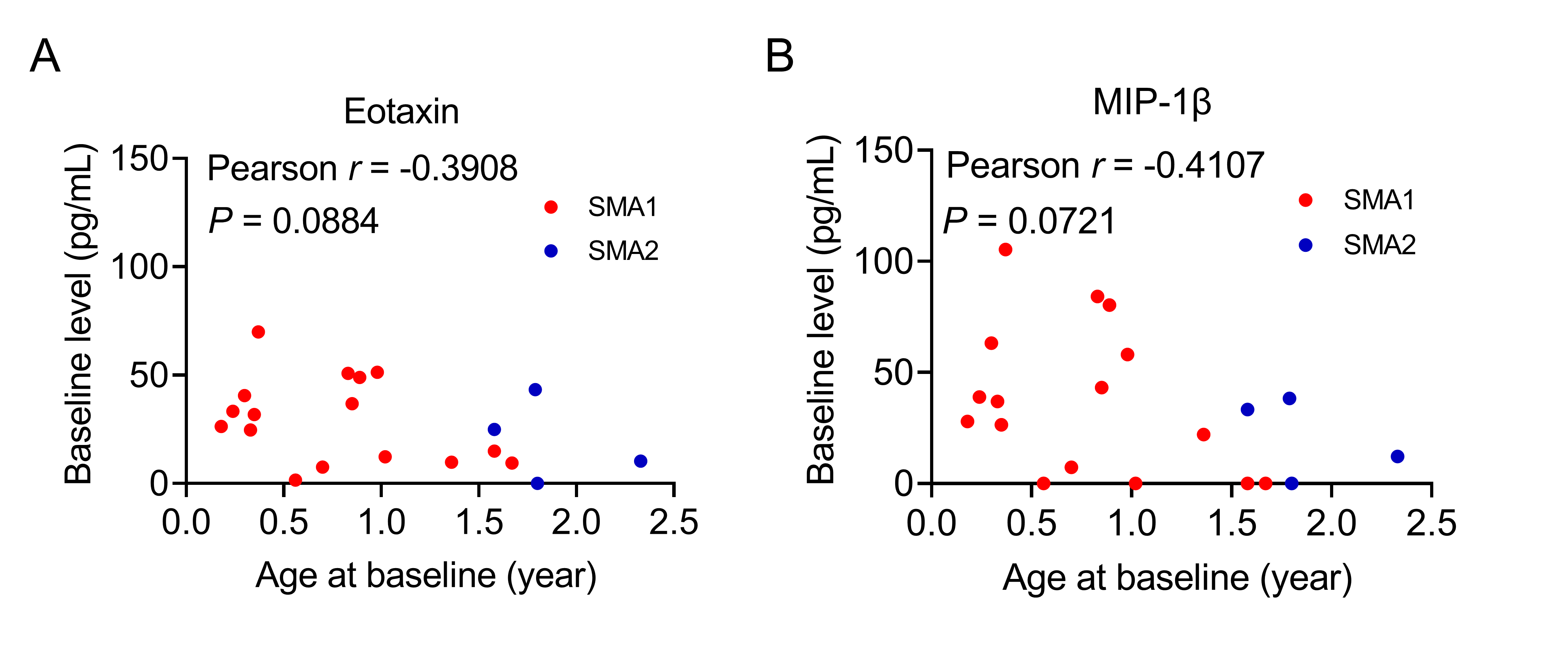


**Supplementary Figure 4.** Correlation analysis of baseline CSF levels of Eotaxin (A) and MIP-1β (B) with the age of patients at the time of baseline CSF sample collection. Pearson’s correlation analysis was performed to assess the correlation.


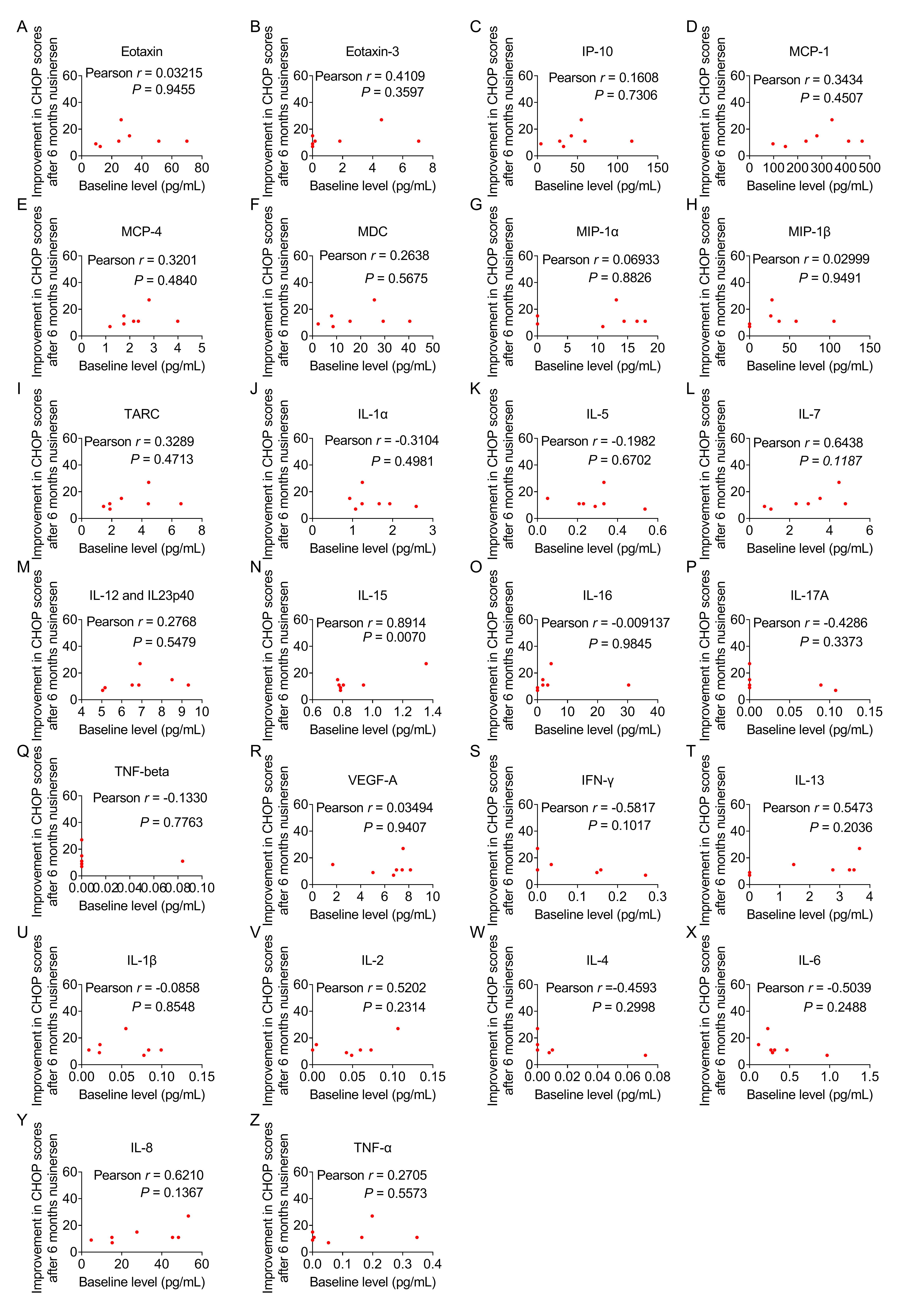


**Supplementary Figure 5. Correlation analysis of baseline levels of neuroinflammatory markers with changes in CHOP motor function scores in SMA1 patients after 6 months of nusinersen treatment**. No significant correlations were detected between the baseline levels of Eotaxin (A), Eotaxin-3 (B), IP-10 (C), MCP-1 (D), MCP-4 (E), MDC (F), MIP-1α (G), MIP-1β (H), TARC (I), as well as cytokines IL-1α (J), IL-5 (K), IL-7 (L), IL-12/IL-23p40 (M), IL-15 (N), IL-16 (O), IL-17A (P), TNF-beta (Q), VEGF-A (R), and proinflammatorys IFN-γ (S), IL-13 (T), IL-1β (U), IL-2 (V), IL-4 (W), IL-6 (X), IL-8 (Y) and TNF-α (Z), with clinical motor function improvement following the therapeutic intervention. Pearson’s correlation analysis was performed.

**Supplementary Figure 6. CSF levels of neuroinflammatory markers during the first 6 months of nusinersen treatment.** No significant differences were detected in the CSF levels of IL-1α (A), IL-1β (B), IL-5 (C), IL-6 (D), IL-7 (E), IL-10 (F), IL-13 (G), IL-15 (H), IL-16 (I), IL-17A (J), Eotaxin-3 (K), TNF-α (L), TNF-beta (M), TARC (N), MCP-4 (O), IP-10 (P), and INF-γ (Q) across any time points during the six-month Nusinersen treatment in patients. Data were analyzed by One-way ANOVA followed by Turkey’s post-hoc test or the Kruskal-Wallis test when the normal distribution assumption was met. In cases where the normal distribution assumption was not met, the Brown-Forsythe and Welch ANOVA tests for nonparametric comparisons were performed.


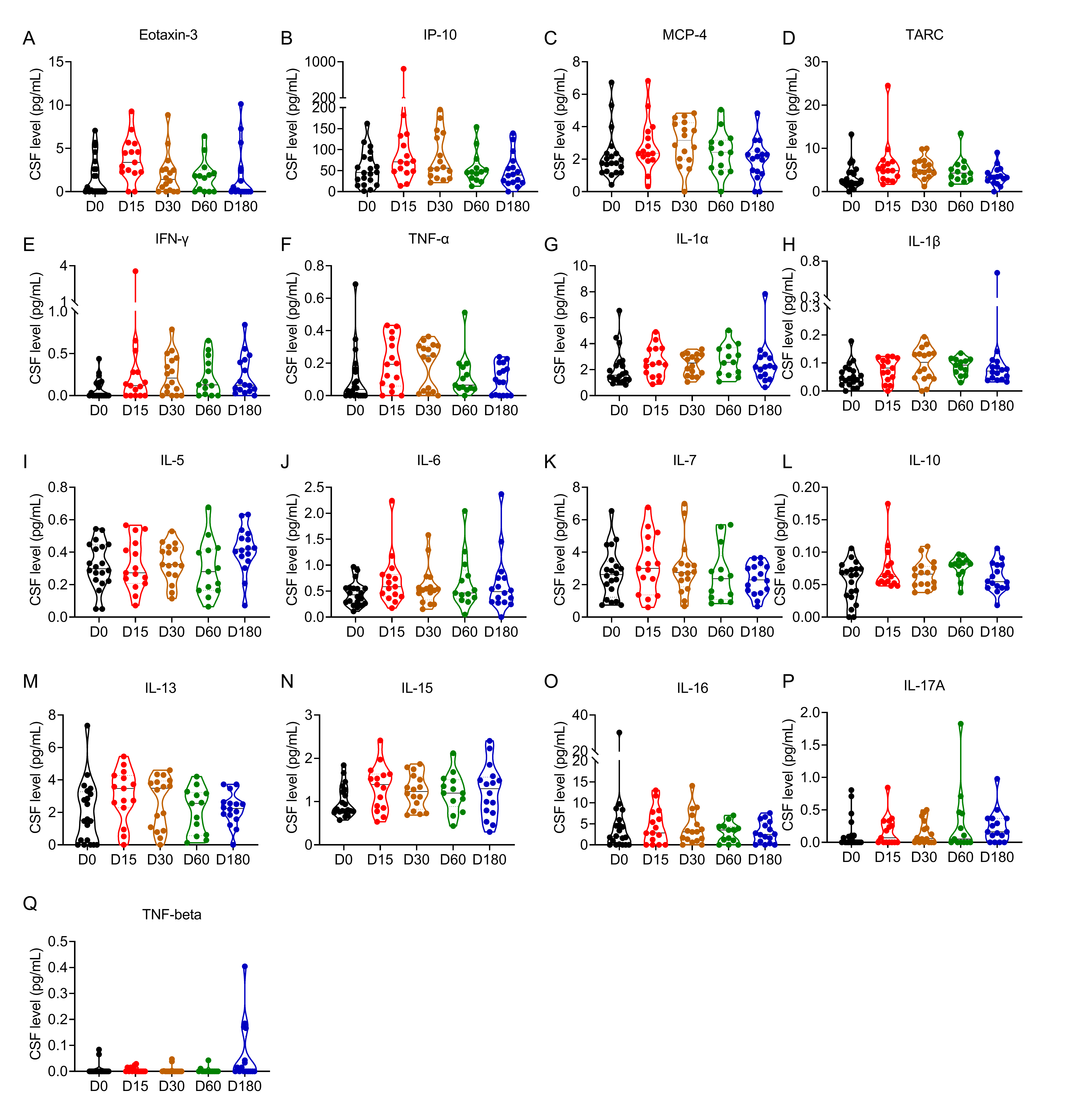


**Supplementary Table 1.** The levels of neuroinflammatory markers in CSF from SMA patients at different time points

|  | **CSF level**  **(pg/mL)** | **D0** | **D15** | **D30** | **D60** | **D180** |
| --- | --- | --- | --- | --- | --- | --- |
| **Chemokine** | Eotaxin | 25.6（10.2-41.3） | 31.8（26.7-49.0） | 24.4（14.3-41.6） | 11.8（10.2-20.2） | 8.2（2.8-14.3） |
|  | Eotaxin-3 | 0.4（0.0-2.8） | 3.4（2.3-5.1） | 1.4（0.1-2.5） | 1.8（0.0-2.2） | 0.1（0.0-2.5） |
|  | IP-10 | 45.6(24.7-79.0) | 71(48.3-121.4) | 56.0(36.5-130.8) | 46.7(32.7-56.3) | 39.6(22.0-61.1) |
|  | MCP-1 | 246.0(190.5-359.8) | 315.5(259.8-439.6) | 514.4(319.7-538.8) | 322.0(292.2-475.7) | 310.1(228.2-348.4) |
|  | MCP-4 | 1.8(1.2-2.4) | 2.4(2.1-3.5) | 3.2(2.0-4.2) | 2.4(1.5-3.0) | 2.1(1.2-2.4) |
|  | MDC | 13.0(7.1-27.3) | 28.0(21.8-41.0) | 23.6(19.8-37.8) | 21.1(11.5-28.0) | 11.6(4.3-14.5) |
|  | MIP-1α | 12.8(9.2-15.5) | 15.4(10.8-20.1) | 13.6(9.7-19.4) | 12.3(5.1-12.6) | 10.6(0.0-13.4) |
|  | MIP-1β | 30.6(5.5-46.9) | 40.0(32.0-46.4) | 34.2(0.9-52.2) | 10.7(0.0-31.0) | 0.0(0.0-20.4) |
|  | TARC | 2.3(1.9-4.4) | 4.9(3.3-6.7) | 5.0(4.2-6.9) | 4.3(2.9-5.6) | 3.3(2.4-4.5) |
| **Cytokine** | IL-1a | 1.6（1.2-2.4） | 2.4（1.6-3.6） | 2.5（1.7-3.2） | 2.6（1.7-3.6） | 2.2（1.6-3.0） |
|  | IL-5 | 0.3（0.2-0.4） | 0.3（0.2-0.4） | 0.3（0.3-0.4） | 0.3（0.2-0.4） | 0.4（0.4-0.5) |
|  | IL-7 | 2.6(1.6-3.2) | 3.0(1.9-4.6) | 2.8(2.1-3.2) | 2.4(1.2-2.9) | 2.3(1.6-3.1) |
|  | IL-12/IL-23p40 | 6.5(4.9-7.9) | 8.2(5.1-9.1) | 6.1(4.8-7.4) | 4.6(3.5-8.1) | 5.6(4.0-6.6) |
|  | IL-15 | 0.8(0.8-1.2) | 1.4(0.9-1.6) | 1.2(0.9-1.5) | 1.2(1.0-1.5) | 1.3(0.8-1.5) |
|  | IL-16 | 1.9(0.1-5.0) | 2.9(0.6-7.0) | 3.1(1.2-5.4) | 3.5(1.1-4.4) | 2.5(0.7-4.8) |
|  | IL-17A | 0.0(0.0-0.1) | 0.1(0.0-0.3) | 0.1(0.0-0.2) | 0.1(0.0-0.4) | 0.2(0.1-0.4) |
|  | TNF-β | 0.000(0.000-0.000) | 0.000(0.000-0.013) | 0.000(0.000-0.000) | 0.000(0.000-0.000) | 0.008(0.000-0.074) |
|  | VEGF-A | 5.4(3.7-7.5) | 7.5(5.2-9.3) | 7.6(6.8-9.7) | 7.8(4.4-8.4) | 8.3(7.5-11.8) |
| **Proinflammatory** | IFN-γ | 0.0(0.0-0.2) | 0.1(0.0-0.3) | 0.2(0.0-0.4) | 0.1(0.0-0.4) | 0.1(0.1-0.4) |
|  | IL-10 | 0.06(0.03-0.07) | 0.06(0.05-0.08) | 0.07(0.05-0.08) | 0.08(0.07-0.09) | 0.05(0.05-0.08) |
|  | IL-13 | 1.5(0.2-3.2) | 3.5(2.4-4.2) | 3.5(2.4-4.2) | 2.6(0.6-3.2) | 2.2(1.8-2.6) |
|  | IL-1β | 0.05(0.01-0.08) | 0.1(0.0-0.1) | 0.1(0.0-0.1) | 0.1(0.1-0.1) | 0.1(0.0-0.1) |
|  | IL-2 | 0.05(0.01-0.08) | 0.12(0.08-0.17) | 0.12(0.06-0.15) | 0.14(0.12-0.15) | 0.12(0.08-0.15) |
|  | IL-4 | 0.00(0.00-0.01) | 0.01(0.00-0.02) | 0.00(0.00-0.02) | 0.01(0.00-0.03) | 0.04(0.01-0.04) |
|  | IL-6 | 0.4(0.3-0.6) | 0.6(0.4-0.8) | 0.5(0.4-0.6) | 0.5(0.4-0.8) | 0.5(0.3-0.8) |
|  | IL-8 | 19.4(14.7-40.5) | 23.6(15.7-45.7) | 32.8(21.5-51.3) | 19.6(18.7-22.5) | 18.1(13.6-23.2) |
|  | TNF-α | 0.0(0.0-0.2) | 0.2(0.1-0.3) | 0.2(0.0-0.3) | 0.1(0.0-0.2) | 0.1(0.0-0.2) |

Neuroinflammatory marker levels in CSF samples from SMA patients at different time points. The values of neuroinflammatory markers measured by the MDS assay in CSF samples collected from SMA patients are presented as median (interquartile range) across different time points.
